# Submillimeter diffusion tensor imaging and late gadolinium enhancement cardiovascular magnetic resonance of chronic myocardial infarction

**DOI:** 10.1186/s12968-016-0317-3

**Published:** 2017-01-11

**Authors:** Farhad Pashakhanloo, Daniel A. Herzka, Susumu Mori, Muz Zviman, Henry Halperin, Neville Gai, David A. Bluemke, Natalia A. Trayanova, Elliot R. McVeigh

**Affiliations:** 1 Department of Biomedical Engineering, Johns Hopkins University, Baltimore, MD USA; 2 Department of Radiology, Johns Hopkins University, Baltimore, MD USA; 3 Department of Medicine, Johns Hopkins University, Baltimore, MD USA; 4 Radiology and Imaging Sciences, National Institutes of Health Clinical Center, Bethesda, MD USA; 5 Departments of Bioengineering, Medicine, Radiology, University of California, 9500 Gilman Drive-MC0412,La Jolla, San Diego, 92093-0412 CA USA

**Keywords:** Myocardial infarction, Fiber structure, Microstructural remodeling, Diffusion tensor imaging, Late gadolinium enhancement

## Abstract

**Background:**

Knowledge of the three-dimensional (3D) infarct structure and fiber orientation remodeling is essential for complete understanding of infarct pathophysiology and post-infarction electromechanical functioning of the heart. Accurate imaging of infarct microstructure necessitates imaging techniques that produce high image spatial resolution and high signal-to-noise ratio (SNR). The aim of this study is to provide detailed reconstruction of 3D chronic infarcts in order to characterize the infarct microstructural remodeling in porcine and human hearts.

**Methods:**

We employed a customized diffusion tensor imaging (DTI) technique in conjunction with late gadolinium enhancement (LGE) cardiovascular magnetic resonance (CMR) on a 3T clinical scanner to image, at submillimeter resolution, myofiber orientation and scar structure in eight chronically infarcted porcine hearts *ex vivo*. Systematic quantification of local microstructure was performed and the chronic infarct remodeling was characterized at different levels of wall thickness and scar transmurality. Further, a human heart with myocardial infarction was imaged using the same DTI sequence.

**Results:**

The SNR of non-diffusion-weighted images was >100 in the infarcted and control hearts. Mean diffusivity and fractional anisotropy (FA) demonstrated a 43% increase, and a 35% decrease respectively, inside the scar tissue. Despite this, the majority of the scar showed anisotropic structure with FA higher than an isotropic liquid. The analysis revealed that the primary eigenvector orientation at the infarcted wall on average followed the pattern of original fiber orientation (imbrication angle mean: 1.96 ± 11.03° vs. 0.84 ± 1.47°, *p* = 0.61, and inclination angle range: 111.0 ± 10.7° vs. 112.5 ± 6.8°, *p* = 0.61, infarcted/control wall), but at a higher transmural gradient of inclination angle that increased with scar transmurality (r = 0.36) and the inverse of wall thickness (r = 0.59). Further, the infarcted wall exhibited a significant increase in both the proportion of left-handed epicardial eigenvectors, and in the angle incoherency. The infarcted human heart demonstrated preservation of primary eigenvector orientation at the thinned region of infarct, consistent with the findings in the porcine hearts.

**Conclusions:**

The application of high-resolution DTI and LGE-CMR revealed the detailed organization of anisotropic infarct structure at a chronic state. This information enhances our understanding of chronic post-infarction remodeling in large animal and human hearts.

**Electronic supplementary material:**

The online version of this article (doi:10.1186/s12968-016-0317-3) contains supplementary material, which is available to authorized users.

## Background

Myocardial infarction (MI) is a major cause of death, affecting millions of people worldwide [1]. The occurrence of MI initiates a complex time-dependent and dynamic process of cardiac remodeling that leads to changes in tissue composition, heart geometry, and organ function [2]. While it is clear that MI can lead to heart failure and arrhythmias [3, 4], the exact link between post-MI structural remodeling and the electromechanical functioning of the heart is not completely understood. Accurate knowledge of infarct structure and fiber orientation remodeling in the intact heart is essential for understanding MI pathophysiology. The need for such data is underscored by the fact that infarct structural remodeling is complex and three-dimensional (3D) in nature, which is reflected in the associated changes in cardiac function. However, there is a paucity of data regarding the detailed three-dimensional scar geometry and MI fiber orientation remodeling in intact large animal and human hearts.

Early renditions of myocardial fiber structure were based on sectioning approaches [5]. These methods produce excellent high-resolution data of local tissue structure [6]; however, combining these measurements together into a registered whole-organ data set, particularly in large animal and human hearts, is extremely difficult. Diffusion Tensor Imaging (DTI) is a non-destructive tool that utilizes the restricted diffusivity of water molecules to assess the tissue microstructure [7]. DTI yields data on the mean diffusivity of water molecules, quantified by Mean Diffusivity (MD), as well as the directional variability of the water diffusion measured by Fractional Anisotropic (FA). Importantly, the principal diffusion eigenvector reflects the mean intravoxel orientation in the tissue. Ventricular fiber maps derived from DTI of formalin-fixed hearts correlate well with histological measurements [8, 9]. DTI has been utilized in infarcted animal and human hearts to characterize structural remodeling using diffusivity measures [10–12], and to assess the remodeling in fiber arrangement at the infarct and in remote locations [13–18]. However, reliable imaging of the fiber orientation at and near the infarct has proven challenging [14], because the chronically infarcted region is often associated with significant wall thinning, and hence a higher image spatial resolution is required to reliably track the fiber angles across the infarcted wall. In addition, the low diffusion anisotropy in the infarct [12, 14] increases the measurement uncertainties in determining the principal eigenvectors of the diffusion tensors [19], particularly in a low image signal-to-noise ratio (SNR) regime [19, 20]. Accurate reconstruction of fiber orientation in infarcted hearts thus necessitates DTI sequences that produce high image spatial resolution and high SNR. Our group has recently developed such methodology that has proven successful in imaging myofiber orientation in the thin atrial walls [21].

The goal of this study was to image the 3D chronic infarct structure at a submillimeter resolution and to provide a systematic analysis of the microstructural remodeling at the infarct. To do so, we employed our previously developed [21] 3D DTI sequence and applied it to image infarcted porcine and human ventricles *ex vivo*. For the infarcted porcine hearts, this technique was used in conjunction with high-resolution T1-weighted late gadolinium enhancement (LGE) cardiovascular magnetic resonance (CMR), to reconstruct the ventricular fiber organization and scar geometry in the same heart at image resolution and SNR higher than previously achieved [14]. The detailed knowledge of infarct microstructure and fiber orientation obtained in this study is expected to enhance our understanding of post-infarction remodeling that underlies rhythm and pump disorders, thus providing impetus to improvements in targeted therapies.

## Methods

### Specimen acquisition and preparation in porcine hearts

Anteroapical infarction was created by occluding the mid–left anterior descending (LAD) coronary artery in female Yorkshire porcine for 120 min using a balloon angioplasty catheter (n = 8). The hearts were excised at least 3.5 months after the induction of the MI (average MI age: 6.7 ± 2.9 months). As the gold standard for MI imaging, a double dose of Gd-DTPA (Magnevist®) was administered (0.2 mmol/kg) through Intravenous (IV) line 20 min before animal sacrifice. Under anesthesia, the animals were injected with heparin to prevent clot formation in the heart. Further, the heart was arrested using potassium chloride (KCl) to avoid contraction. Excision was performed under 5 min after the animal sacrifice, and the ventricles were filled with rubber (Task5™) to keep the heart in the natural unloaded shape. To avoid specimen dehydration and susceptibility artifacts resulting from the tissue-air interface, the hearts were submerged in perfluorocarbon (Fluorinert-77, 3 M) prior to subsequent imaging. To serve as controls, 4 normal porcine hearts were harvested from animals with no prior MI and prepared in a similar fashion. The weight of the animals at the time of the first procedure (MI induction for the infarcted hearts and the heart harvest for the control hearts) was 50 ± 18 kg, and at the time of harvest for the infarcted hearts was 121 ± 62 kg. Porcine age was approximated as 4 months at the time of the first procedure.

### Late gadolinium enhancement CMR

For the infarcted porcine hearts, LGE was performed using a 3D T1-weighted Gradient Echo sequence with radiofrequency (RF) spoiling, approximately 1 h after the excision, to image the whole heart. Imaging was performed with the following parameters: acquired resolution = 0.25 × 0.25 × 0.50 mm^3^, typical field of view = 110 × 110 × 140 mm^3^, echo time (TE) = 2.3 ms, repetition time (TR) = 12 ms, flip-angle = 15°, number of averages = 3, scan duration = 1 h.

### Diffusion tensor imaging

The specimens were fixed in 10% buffered formaldehyde more than 40 days prior to DTI acquisition. The time from harvest to fixation was about 2–3 h. Our 3D Fast Spin Echo DTI sequence developed previously [21] was used on a 3T clinical system (Achieva TX, Philips Healthcare, Best, The Netherlands) to image the whole hearts *ex vivo* (n = 4 control porcine, n = 8 infarcted porcine and one human heart). Imaging parameters were: TE = 63 ms, TR = 504 ms, bandwidth = 290.0 Hz/pixel, number of echoes = 2, diffusion gradients duration = 22.8 ms, time gap between diffusion pulses = 12.6 ms, maximum gradient strength = 60 mT/m, RF coil: Philips 8-channel head coil, number of diffusion encoding directions = 15, maximum b-value = 800 s/mm^2^, typical field of view = 110 × 115 × 130 mm^3^, acquired voxel dimension = 0.6 × 0.6 × 1.2 mm^3^, reconstructed voxel dimension (using zero-padding) = 0.4 mm^3^, and total scan duration ~ 42 h.

### Tensor calculation and tissue segmentation

**Fig. 1:**
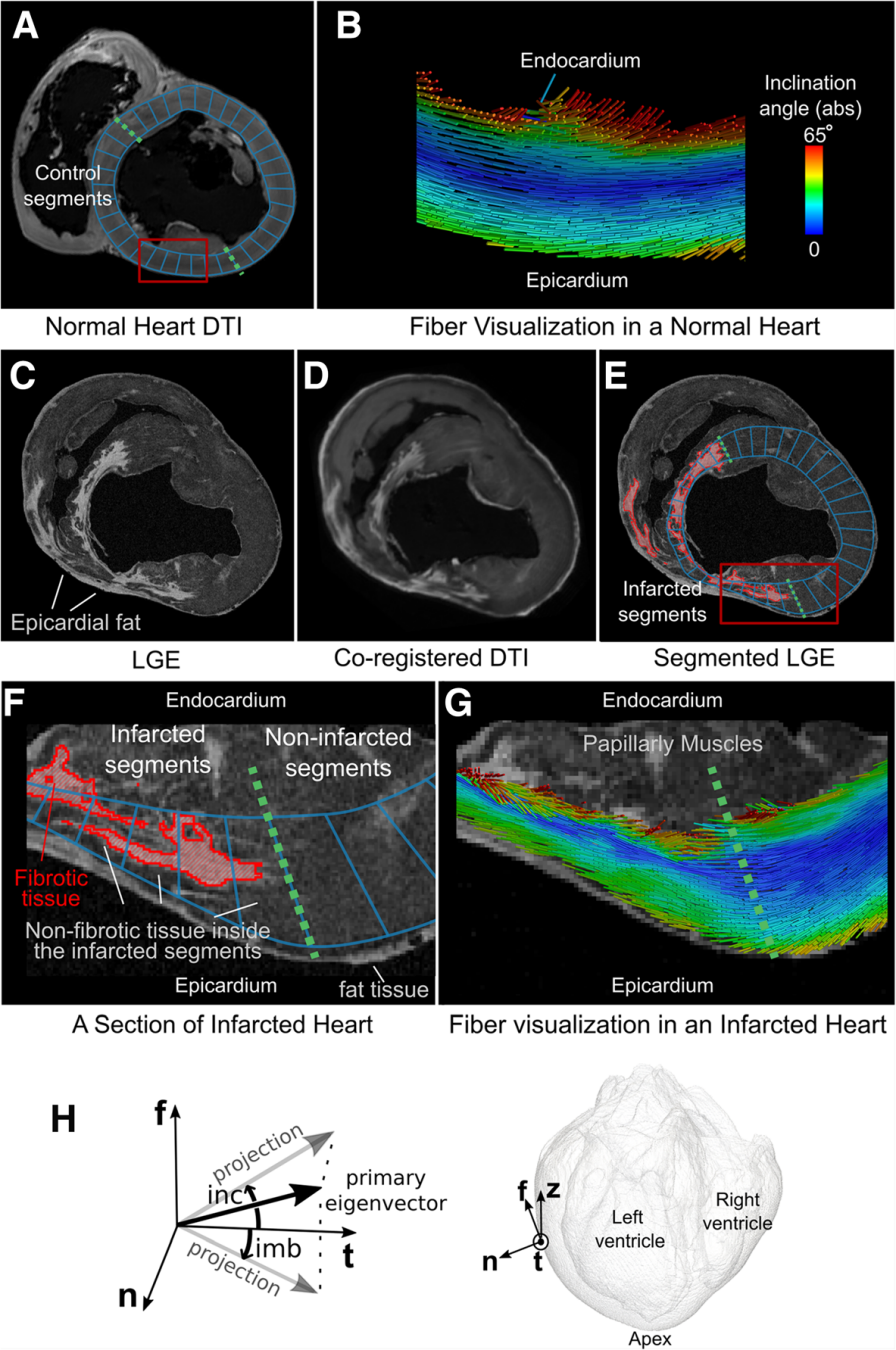
Structural remodeling in the infarcted heart. **A** A short-axis slice of a normal heart with left ventricle (*LV*) segments in *blue*. The two dashed green lines delineate the control segments. **B** Visualization of eigenvectors in a section (*red box* in **A**) of the anterior wall. Fibers are color-coded based on absolute values of inclinations angles, i.e. *blue* signifies circumferential fiber orientation. **C** Short-axis LGE image of an infarcted porcine heart and **D** the co-registered non-diffusion-weighted image. **E** The same slice as in **C** and **D** but with LV segments and fibrosis mask overlaid in blue and red, respectively. **F** Zoomed-in view of the red box in (**E**) highlighting fibrotic and non-fibrotic tissues inside infarcted segments. *Green dashed* line demarcates infarcted and non-infarcted segments. **G** Fiber visualization in the region shown in **F** highlighting the transition from non-infarcted to infarcted tissue, with the color-coding as in **B**. **H** Measurement of inclination (*inc*) and imbrication (*imb*) angles of primary eigenvector in a local coordinate system (*n, t, f*) that is tangential to the endocardial surface – see Methods for the definitions

Raw MRI data were exported from the scanner and a customized image reconstruction was performed offline using MATLAB (The MathWorks Inc., Natick, MA). Diffusion tensors were calculated using DTI Studio [22]. Next, diffusion eigenvectors and eigenvalues were calculated in the normal porcine hearts (Fig. 1A, B). For the infarcted porcine hearts, the DTI volumes were first co-registered to T1-weighted LGE data (Fig. 1C) using 3D affine transformations and the diffusion tensors were transformed accordingly. The spatial registration enabled us to reconstruct both fiber structure and scar geometry in the same coordinate system in each MI heart (Fig. 1E), and to correct for the slight shrinkage of heart tissue due to the fixation process. Further, the left ventricular (LV) endocardial and epicardial surfaces were contoured to delineate the LV in each heart. In this process, papillary muscles and trabecular structures were excluded (Fig. 1A, E). Next, an Otsu thresholding algorithm [23] (n = 2) followed by a level-set segmentation was applied in Seg3D software (http://www.seg3d.org) to the LGE images to classify ventricular tissue into two regions, fibrotic (enhanced) and non-fibrotic (Fig. 1E).

### Fiber angle measurement and visualization

In all the hearts, the primary diffusion eigenvector angles were measured in a local coordinate system tangential to the LV endocardial surface (Fig. 1H). The unit vectors of the three orthogonal axes of this coordinate system, *n*, *t* and *f*, were defined as follows: *n*: the normal vector to the endocardial surface, *t*: circumferential vector, such that *t* = *z* × *n* (where *z* is the longitudinal unit vector directed from apex to base), and *f* = *n* × *t*. The definition of this coordinate system guarantees that *t* and *f* are tangential to the heart surface, even in the case that *n* does not lie within the short-axis plane of the heart due to the heart curvature. As shown in Fig. 1H, the orientation of the primary eigenvector was uniquely determined by two fiber angles measured in this coordinate system. The inclination angle was defined as the angle between the projection of the primary eigenvector onto the tangential plane (defined by *t* and *f*) and the local circumferential vector (*t*). Likewise, the imbrication angle was defined as the angle between the circumferential vector (*t*) and the projection of the eigenvector onto the plane defined by *t* and *n*. In a normal LV wall, the inclination angle changes smoothly from the epicardium with fibers having negative inclination angle (left-handed fibers) to the endocardium with fibers having positive inclination angle (right-handed fibers). The circumferentially running fibers at the midwall have an inclination angle close to zero. The imbrication angle is, on average, close to zero in a normal LV wall. Inclination and imbrication angles were measured at all myocardial voxels throughout each heart. In all the figures in this study, the vector field associated with the primary eigenvector was visualized in TrackVis [24]. This was performed by representing each eigenvector by a small line/tube of fixed dimensions that is located at that voxel and aligns with the primary eigenvector. The fiber orientations were color-coded using the inclination angle (Fig. 1B).

### Analysis of regional remodeling: definition of LV segments in porcine hearts

To characterize the 3D regional structural remodeling in MI, LVs of normal and infarcted porcine hearts were partitioned into small transmural segments, as shown in Fig. 1A, E. For each short-axis slice of 1.2 mm thickness (excluding apical slices), a polar coordinate system was defined with an origin at the center of the blood mass. In this coordinate system, each LV was divided circumferentially into 36 segments of 10 angular width per segment. This resulted in about N ~ 1000 segments per heart.

Infarcted segments were defined as the LV segments that contained fibrotic tissue on the LGE images (Fig. 1E, F). These segments were located at the anteroseptal wall, consistent with the LAD infarction protocol. The corresponding segments from the same anatomical locations in the normal hearts were selected as control segments (spanning 180° of the LV anteroseptal wall, as delineated by the green dashed lines in Fig. 1B). Due to the presence of viable tissue surrounding the scar and the complex scar geometry, the transmural infarcted segments could contain both fibrotic and non-fibrotic tissues (Fig. 1F).

### Analysis of regional remodeling: quantification of local segment structure in porcine hearts

Subsequent to defining LV transmural segments, voxel data from individual infarcted and control segments were analyzed to determine local fiber angles, wall thickness, and fibrosis extent. The following metrics were defined for each segment: *wall thickness*, the wall thickness of the segment; *scar transmurality*, the ratio of number of fibrosis voxels (as obtained from the LGE data) in a segment to the total number of voxels in a segment; *slope and intercept*, the slope and the intercept of the linear regression model fitted to the inclination angle transmural profile data (angle vs. depth of the wall as measured from the epicardium); *r* ^*2*^, the coefficient of determination (R-squared) of the regression model of the inclination angle profile; (*epi-to-endo*) *inclination angle range*, the range of inclination angle within each segment as calculated from the [1–99]% range of inclination angle distribution; *imbrication angle mean*, the average of the imbrication angles within a segment; *left-handed ratio (LH ratio),* the ratio of the number of voxels in which inclination angle < −15° to the total number of voxels within a segment; *circumferential ratio*, same ratio but calculated for the voxels with −15° < inclination angle < 15°; and *right-handed ratio (RH ratio)*, same ratio but for voxels with inclination angle > 15°. We also quantified local intervoxel incoherency in eigenvector arrangement corresponding to both the inclination and imbrication angles (see the Additional file 1: Methods for the definition).

### Statistical analysis

Statistics were performed on the quantities obtained from the infarcted and control segments throughout the porcine hearts to study the microstructural differences between these regions. Non-parametric Wilcoxon rank-sum test was performed between the averaged segment values from the infarcted (n = 8) and control (n = 4) hearts (per heart statistics). The same statistics was performed on the pooled voxel or segment data (per voxel/segment statistics). All the data is presented as mean ± standard deviation (STD). The statistics presented in the text of the manuscript are from the per heart analysis, unless stated otherwise. Further, Pearson correlations were calculated to study the pair-wise associations of slope, inclination angle range, wall thickness, and scar transmurality in the infarcted segments from porcine hearts.

### Imaging and analysis of infarcted human heart specimen

In addition to the porcine hearts, an intact human heart was procured through the National Disease Research Interchange (NDRI, Philadelphia, PA). This heart was from a donor with a history of MI (93 years old female). The human heart specimen had already undergone fixation process at the time of acquisition and hence was not imaged using LGE-CMR (fixation time from harvest: 7 h). It was imaged using the same diffusion tensor imaging sequence at the resolution of 0.5 × 0.5 × 1.0 mm^3^. To examine infarct remodeling in the human heart, fiber angle measurement and eigenvector visualization were performed in the same way as in the porcine hearts.

## Results

### LV characteristics of porcine hearts

The eight infarcted porcine hearts demonstrated significant global remodeling with changes in LV mass and LV blood volume (LV mass: 121 ± 47 g in MI vs. 70 ± 9 g in normal hearts, LV blood volume: 81 ± 26 cm^3^ in MI vs. 41 ± 25 cm^3^ in normal hearts). The normal hearts had an average wall thickness of 7.2 ± 1.2 mm; by comparison, the average wall thickness in the infarcted porcine hearts was 5.1 ± 1.2 mm for the infarcted regions and 9.5 ± 1.8 mm otherwise. The scar volume comprised 10.1 ± 7.6% of the LV myocardial volume.

### Measures of diffusivity and anisotropy in porcine hearts

**Fig. 2:**
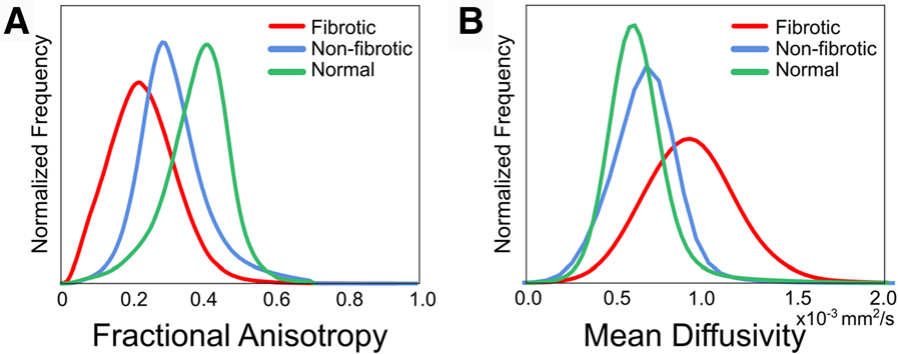
Pooled distributions of diffusion scalars. **A** Fractional Anisotropy and **B** Mean Diffusivity of voxels in the fibrotic and non-fibrotic regions from infarcted hearts, and in control hearts (*green*). The histograms were normalized such that they have the same area under the curve

The mean SNR in non-diffusion-weighted image was 122 ± 11 for control, 184 ± 13 for fibrotic, and 113 ± 15 for non-fibrotic remote regions (see Additional file 1: Methods for the description of the SNR measurements). Diffusion scalar values in fibrotic tissue were compared to both those in non-fibrotic tissue from infarcted hearts and to those from control hearts. Figure 2 presents the pooled distributions of FA and MD, and Table 1 summarizes the diffusivity measures of FA, MD, and the diffusion eigenvalues in fibrotic and non-fibrotic tissues in the infarcted hearts, and in the normal myocardial tissue from the control hearts. Fibrotic tissue exhibited a lower mean FA value (0.24 ± 0.04) compared to non-fibrotic (0.33 ± 0.06, *p* = 0.003) and normal tissue (0.37 ± 0.04, *p* = 0.011). The MD value was higher in fibrotic tissue (9.08 ± 0.72 × 10^−4^ mm^2^/s) as compared to non-fibrotic (6.57 ± 0.92 × 10^−4^ mm^2^/s, *p* < 0.001) and normal tissue (6.33 ± 0.41 × 10^−4^ mm^2^/s, *p* = 0.011). In addition, the pooled distributions of diffusivity measures (Fig. 2B) showed that the fibrotic tissue had a greater dispersion in diffusion eigenvalues than non-fibrotic and normal tissues (see also Additional file 1: Figure S3). Despite the lower diffusion anisotropy observed in the infarct, the FA inside the majority of the infarct was much greater than that of an isotropic liquid (0.027 ± 0.007 – Additional file 1: Figure S2). The histology of infarcted region using Trichrome staining demonstrated the presence of highly aligned collagen fibers inside the fibrotic tissue that could be the main source of anisotropy in the infarct (see Additional file 1: Figure S5 for the results on histology).

**Table 1:** Diffusion scalar measurements in fibrotic and non-fibrotic tissues in the infarcted hearts, and the normal tissue

		Mean ± STD			*P*-values		
	Quantity	Fibrotic	Non-fibrotic	Normal	Fibrotic vs. Non-fibrotic	Fibrotic vs. Normal	Non-fibrotic vs. Normal
Per heart statistics	e_1_ (×10^−4^ mm^2^/s)	11.11 ± 0.64	8.87 ± 0.93	8.95 ± 0.42	<0.001	0.007	0.734
	e_2_ (×10^−4^ mm^2^/s)	9.03 ± 0.71	6.06 ± 0.92	5.66 ± 0.37	<0.001	0.007	0.308
	e_3_ (×10^−4^ mm^2^/s)	7.09 ± 0.88	4.77 ± 0.94	4.39 ± 0.54	0.001	0.007	0.308
	MD (×10^−4^ mm^2^/s)	9.08 ± 0.72	6.57 ± 0.92	6.33 ± 0.41	<0.001	0.007	0.308
	FA	0.24 ± 0.04	0.33 ± 0.06	0.37 ± 0.04	0.003	0.011	0.126
Per voxel statistics	e_1_ (×10^−4^ mm^2^/s)	11.25 ± 2.16	9.01 ± 1.52	8.89 ± 1.14	<0.001	<0.001	<0.001
	e_2_ (×10^−4^ mm^2^/s)	9.23 ± 2.41	6.25 ± 1.53	5.63 ± 1.22	<0.001	<0.001	<0.001
	e_3_ (×10^−4^ mm^2^/s)	7.25 ± 2.47	4.95 ± 1.45	4.35 ± 1.19	<0.001	<0.001	<0.001
	MD (×10^−4^ mm^2^/s)	9.24 ± 2.26	6.74 ± 1.42	6.29 ± 1.09	<0.001	<0.001	<0.001
	FA	0.23 ± 0.09	0.31 ± 0.09	0.37 ± 0.09	<0.001	<0.001	<0.001

e_1_-e_3_: Primary, secondary and tertiary diffusion eigenvalues respectively. Wilcoxon rank-sum test was performed using (top) per heart statistics (n = 8 infarcted and n = 4 control hearts), and (bottom) pooled voxel data

*Abbreviaions*: *MD* mean diffusivity, *FA* fractional anisotropy

### Diffusion eigenvector orientation in porcine hearts

**Fig. 3:**
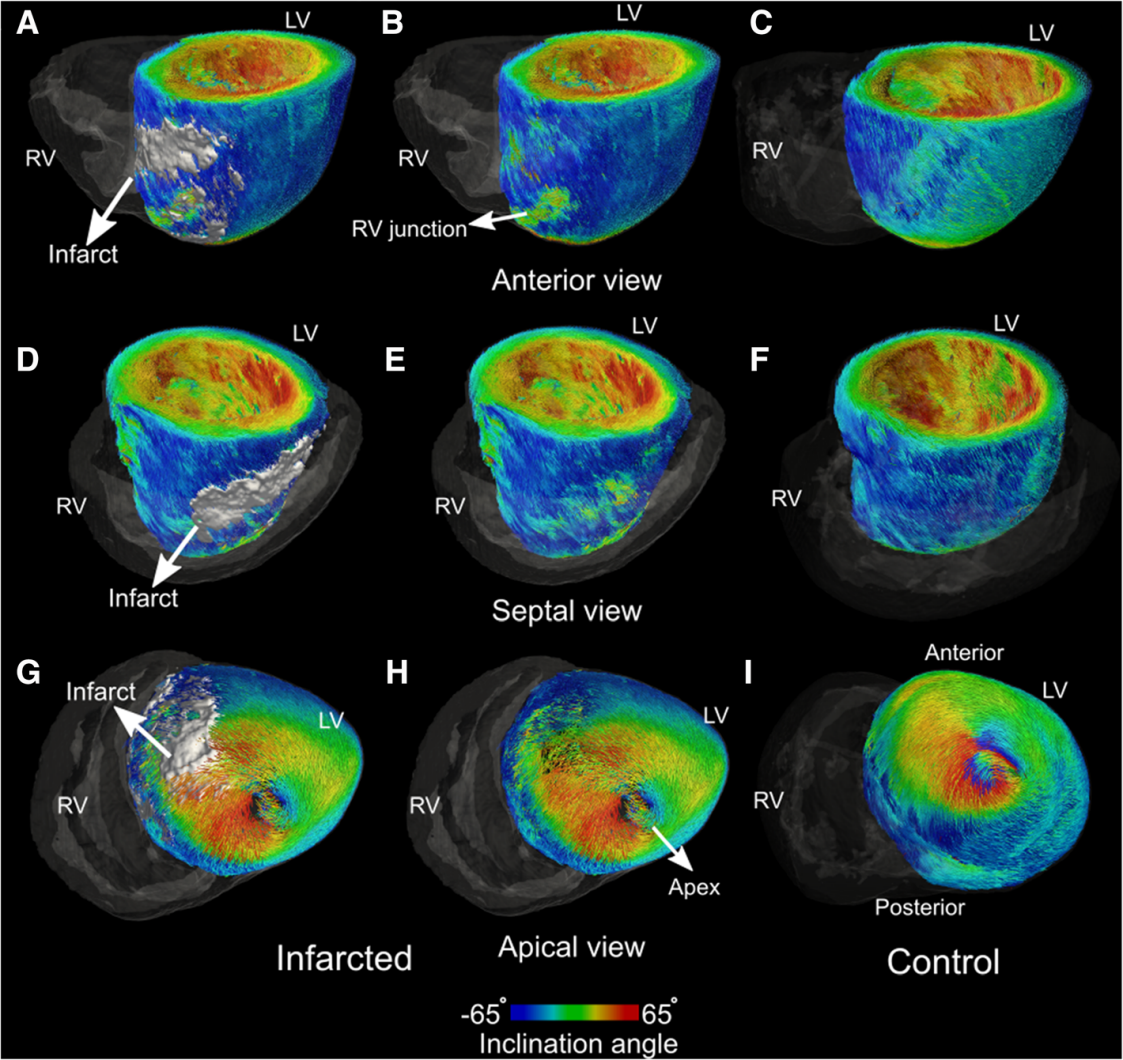
3D visualization of primary eigenvectors in the left ventricle of an infarcted and a control porcine heart. **A**-**C** Anterior view, **D**-**F** Septal view and **G**-**I** Apical view. The fibers are color-coded based on the inclination angle. The transparent gray volume represents the right ventricle. The infarct volume is reconstructed from LGE-MRI and is rendered in gray in **A**, **D**, **G**. The middle panels **B**, **E**, **H** show the same views but without the infarct volumes, to visualize the underlying eigenvectors in the infarct﻿. Similar views of a control heart are shown in the right panels (**C**, **F**, **I**)

The 3D organization of the primary eigenvectors in the LV of an infarcted and a control porcine heart is presented in Fig. 3 with anterior (A, B, C), septal (D, E, F) and apical (G, H, I) views. The left and middle columns demonstrate three views of infarcted heart and the right column presents similar views of the normal heart. The 3D infarct geometry (grey volume) as reconstructed from the LGE is overlaid over the primary eigenvector field in the left column (Fig. 3A, D, G). As the figure demonstrates, the epicardial vectors at the infarct and in its surroundings are, on average, characterized with the original left-handed orientation (i.e. negative inclination angle). This was a consistent finding among the eight infarcted porcine hearts.

**Fig. 4:**
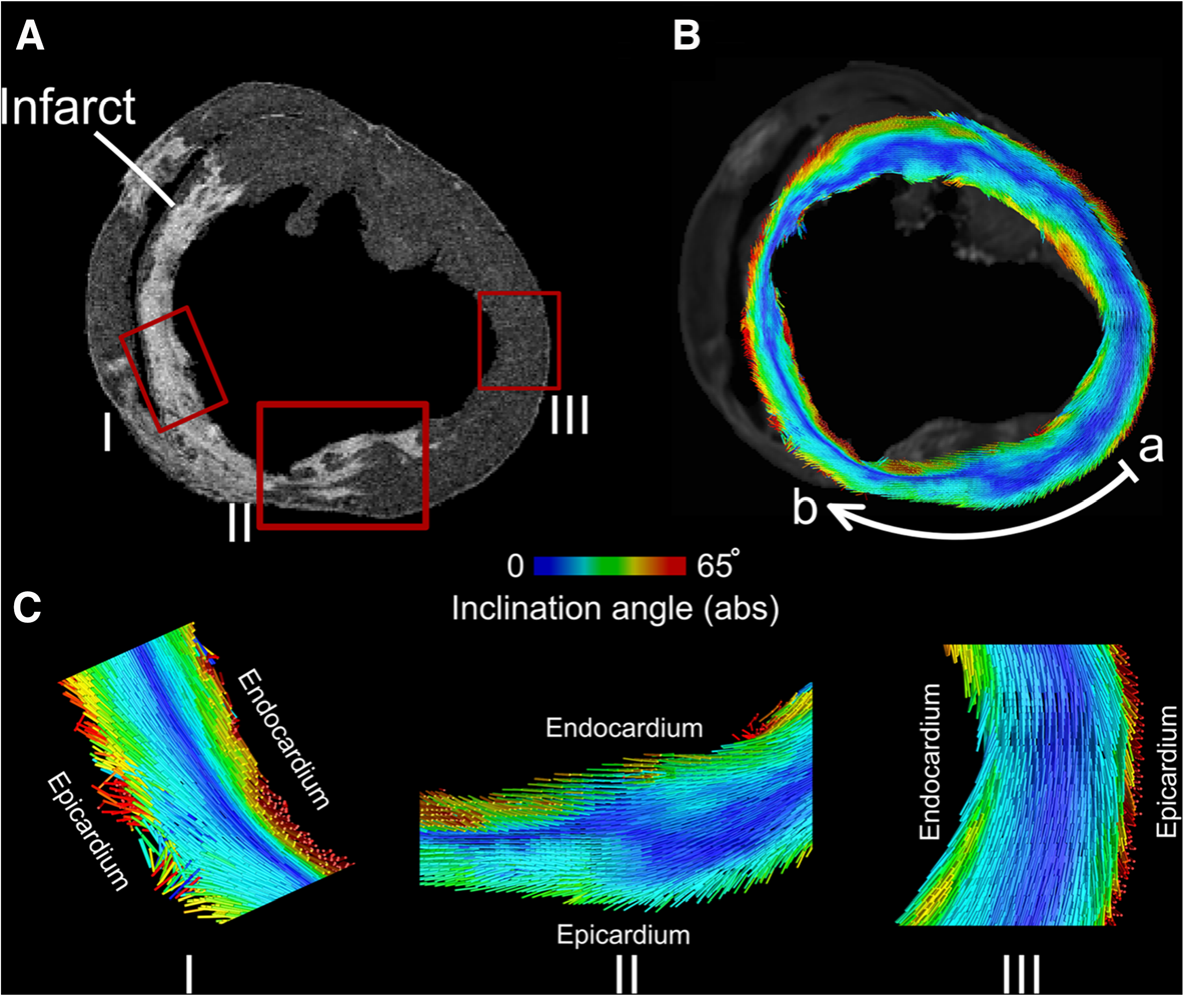
Visualiation of primary eigenvectors in a short-axis slice of an infarcted porcine heart. **A** LGE image showing enhanced image intensity in the infarcted region. **B** Map of left ventricle primary eigenvectors (*same view as* in **A**) color-coded by the absolute values of the inclination angle. The white arc delineates an anterior region of LV that spans from a non-infarcted region (*a*) to an infarcted region (*b*) - see Fig. 5. **C** Eigenvector visualization in three regions (*red boxes* in *a*). I: Infarcted septal wall, II: transition zone between infarcted and non-infarcted portions of the anterior wall, and III: non-infarcted region in the lateral wall

In Fig. 4, the primary eigenvectors are visualized in a short-axis slice of an infarcted heart. The infarct is identifiable by the enhanced area in the corresponding LGE image (Fig. 4A). Fig. 4B shows the primary eigenvectors visualization in the same slice with color-coding based on the inclination angle. Three representative regions of interest have been selected in Fig. 4A and their corresponding eigenvectors are presented in Fig. 4C: (I) an infarcted wall, (II) a transition region between the infarcted and non-infarcted sections of the wall, and (III) a region remote from the infarct. The thinned infarcted wall (I) demonstrates the presence of epicardial, circumferential (blue band) and endocardial primary eigenvectors that have a higher inclination angle transmural gradient across the wall when compared to the section remote from the infarct (III). The middle panel in Fig. 4C (II) presents the transition of eigenvectors from a non-infarcted region with a lower transmural angle gradient to the infarcted region that has a higher angle gradient. The deflection of the circumferential blue band toward the endocardium suggests that there is an immediate increase in the proportion of the left-handed vectors in the infarcted wall (LH-ratio). However, this observation was not consistent across all the transition zones in the porcine hearts as we also observed transition zones that had no deflection of the blue band toward the endocardium. An example of such transition zone is presented in Fig. 1G.

**Fig. 5:**
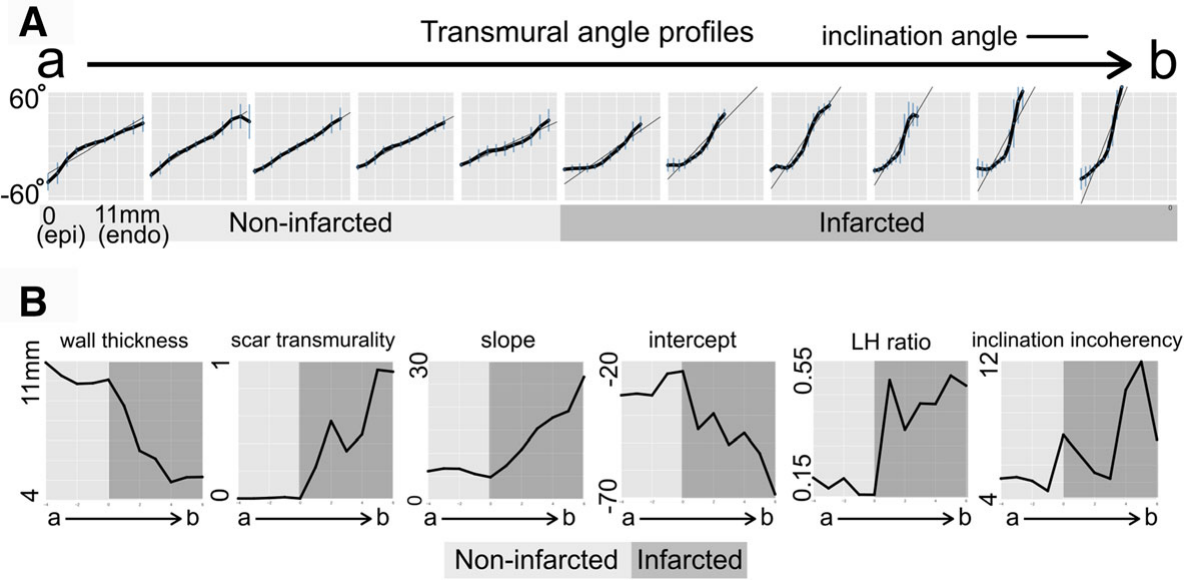
Structural remodeling in a section of the left ventricle wall from Fig. 4. **A** Inclination angle profiles for 11 consecutive segments corresponding to the region delineated by the white arc in Fig. 4B. The horizontal axis of each plot represents wall depth as measured from the endocardium. **B** Structural metrics for the segments in panel **A** plotted as a function of segment location (*a: outside, b: inside the infarct*). Dark areas in each plot denote infarcted segments

Figure 5 presents the quantification of the inclination angle profiles for the same slice in the heart in Fig. 4. In Fig. 5A, the profiles of the inclination angle vs. the wall depth have been plotted for 11 consecutive segments covering the non-infarcted and infarcted regions in the anterior wall (points a to b in Fig. 4B). These profiles reveal an increase in the transmural gradient of the inclination angles in the thinned wall of the infarct, confirming the observation in Fig. 4C. The corresponding structural metrics for each segment are plotted in Fig. 5B and demonstrate an increase in the slope and a decrease in the intercept of the fitted linear models in the infarcted segments. In addition, the LH-ratio increases in the infarcted segments (Fig. 5B). Finally, the infarcted segments exhibit an increase in local incoherency of inclination angles as indicated by the larger error bars (higher variance) in the angle profiles (Fig. 5A, B). Although trabeculae and papillary muscles were excluded from the LV segmentation, we found a significant preservation of these structures at the anterior and septal regions of the infarcted walls in all the hearts. They had extreme values of inclination angles (closer to −90 or 90°), indicating apex-to-base orientation (see Additional file 1: Figure S4 for an example of the presence of papillary muscle fibers at the infarct).

Characteristics of the microstructural remodeling in the infarcted segments are presented in Table 2. The average and standard deviation structural metrics values were computed over ~3300 infarcted and ~1800 control segments from n = 8 infarcted and n = 4 control hearts, and the statistics were performed on the pooled segment data as well as per heart average. The per segment analysis led to statistical significance between the control and infarcted segments in all the parameters due to a larger sample size (and hence higher statistical power); however, the results from the two methods were consistent. The results show that the epi-to-endo range of inclination angle is predominantly preserved inside the infarcted segments (per heart analysis: 111.0 ± 10.7° infarcted vs. 112.5 ± 6.8° control, *p* = 0.610) and that on average, the eigenvectors run parallel to the wall with a small mean imbrication angles (1.96 ± 11.03° infarcted vs. 0.84 ± 1.47° control, *p* = 0.610). In addition, there is an increase in both the inclination incoherency (10.05 ± 1.42° infarcted vs. 5.63 ± 0.56° control, *p* < 0.01) and the imbrication incoherency metrics (8.62 ± 1.77° infarcted vs. 4.83 ± 0.58° control, *p* < 0.01) at the infarcted regions. The transmural slope of the inclination angle showed an average increase inside the infarcted segments (21.6 ± 7.0 °/mm infarcted vs. 15.7 ± 1.1 °/mm control, *p* = 0.126) with a lower r-squared of the linear regression fit in the infarcted segments (0.69 ± 0.05 infarcted vs. 0.93 ± 0.03 control, *p* = 0.007). When only the infarcted segments with a higher range of scar transmurality [0.67–1.00] compared to the control segments, the increase in slope was more substantial, and statistically significant (29.7 ± 9.8 °/mm, *p* = 0.04, see Additional file 1: Table S1), confirming our observation from Fig. 4. Additionally, in comparison to the control segments, the infarcted segments demonstrated a 31% increase in LH-ratio (0.51 ± 0.08 infarct vs. 0.39 ± 0.05 control, *p* = 0.017), as well as a 13% decrease in circumferential-ratio (0.26 ± 0.06, *p* = 0.174) and a 26% decrease in RH-ratio 0.23 ± 0.06, *p* =0.041).

**Table 2:** Structural metrics in infarcted and control segements

	Per heart statistics			Per segment statistics		
Quantity	Infarcted segments	Control segments	*P*-value	Infarcted segments	Control segments	*P*-value
Wall thickness (mm)	5.5 ± 1.1	7.0 ± 0.9	0.041	5.5 ± 2.9	7.0 ± 1.5	<0.001
Scar transmurality	0.42 ± 0.12	NA	NA	0.44 ± 0.30	NA	NA
Inclination angle range (°)	111.0 ± 10.7	112.5 ± 6.8	0.610	109.9 ± 26.1	112.0 ± 15.4	<0.001
Slope (°/mm)	21.6 ± 7.0	15.7 ± 1.1	0.126	21.4 ± 18.7	15.7 ± 3.7	<0.001
Intercept (°)	−60.4 ± 9.1	−54.0 ± 5.3	0.396	−60.9 ± 28.6	−53.9 ± 13.4	<0.001
r^2^	0.69 ± 0.05	0.93 ± 0.03	0.007	0.69 ± 0.23	0.93 ± 0.07	<0.001
Imbrication angle mean (°)	1.96 ± 11.03	0.84 ± 1.47	0.610	1.08 ± 16.63	0.86 ± 8.39	<0.001
Inclination incoherency (°)	10.05 ± 1.42	5.63 ± 0.56	0.007	9.61 ± 3.19	5.70 ± 1.31	<0.001
Imbrication incoherency (°)	8.62 ± 1.77	4.83 ± 0.58	0.007	8.14 ± 3.67	4.85 ± 1.85	<0.001
LH-ratio	0.51 ± 0.08	0.39 ± 0.05	0.017	0.52 ± 0.22	0.39 ± 0.14	<0.001
Circumferential-ratio	0.26 ± 0.06	0.30 ± 0.04	0.174	0.26 ± 0.17	0.30 ± 0.10	<0.001
RH-ratio	0.23 ± 0.06	0.31 ± 0.03	0.041	0.21 ± 0.18	0.31 ± 0.11	<0.001

Wilcoxon rank-sum test was performed between the infarcted and control segments using (left) per heart statistics (n = 8 infarcted and n = 4 control hearts), and (right) per segment statistics (n_infarcted_ ~3300, n_control_ ~1800). The values are represented as mean ± STD

**Fig. 6:**
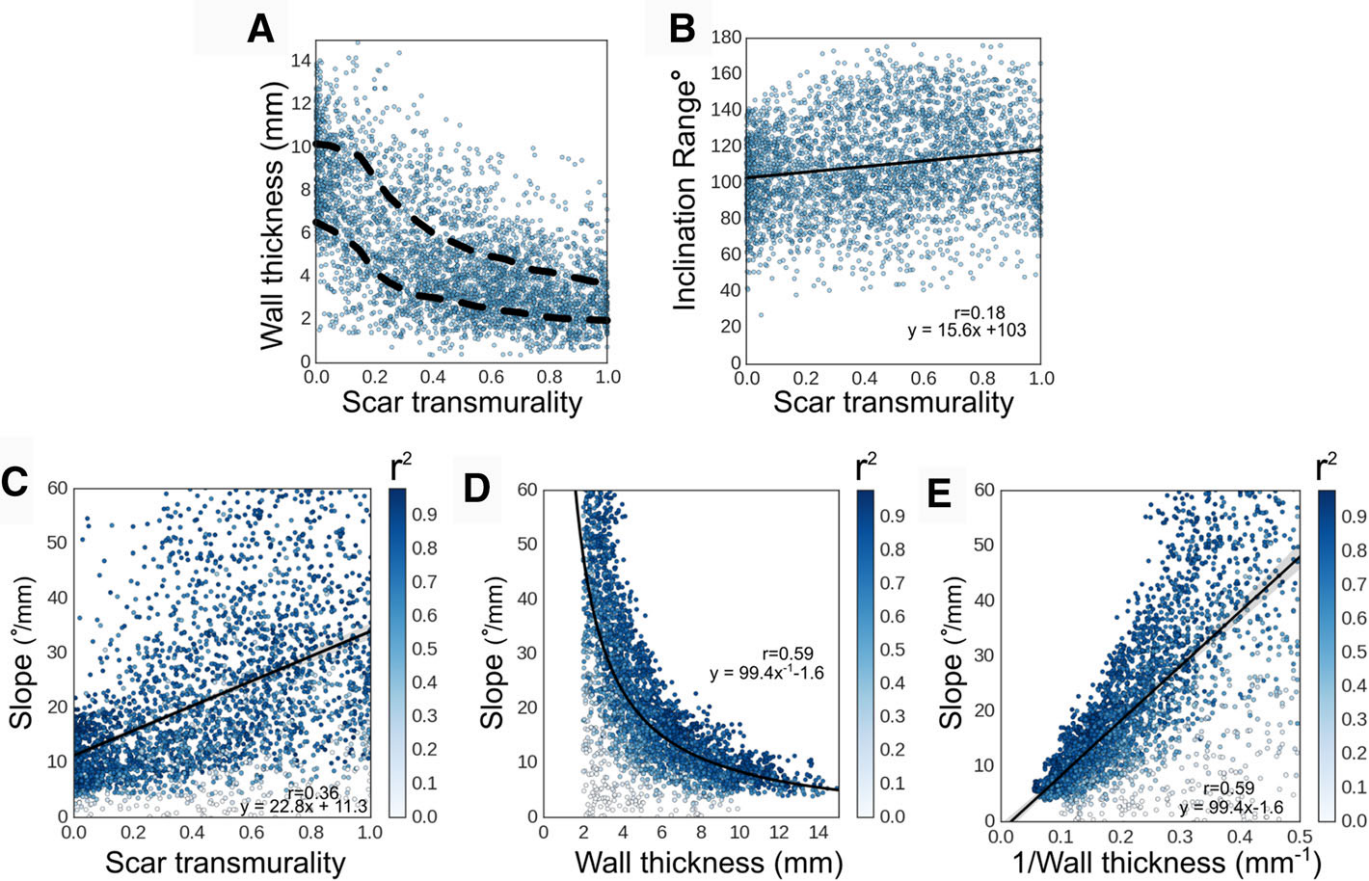
Associating the level of structural remodeling with infarct extent. The scatterplots present data from ~ 3700 infarcted segments. In **A**, *dashed* lines demarcate the 25–75 percentile region. In **B**-**E** *solid* lines represent models fitted to the data. Points in **C**-**E** are color-coded based on r^2^ of the fit for calculation of slope for each data point

Figure 6A presents the association of wall thickness with scar transmurality, as obtained from all the infarcted segments. This figure demonstrates a non-linear decreasing trend of wall thinning as a function of scar transmurality. The scatter plot in Fig. 6B shows that the inclination angle range correlates weakly with scar transmurality (correlation coefficient r = 0.18). Further, slope correlated with scar transmurality (r = 0.36) and importantly, it demonstrated a stronger linear correlation with the inverse of wall thickness (r = 0.59, Fig. 6D, E) than scar transmurality.

**Fig. 7:**
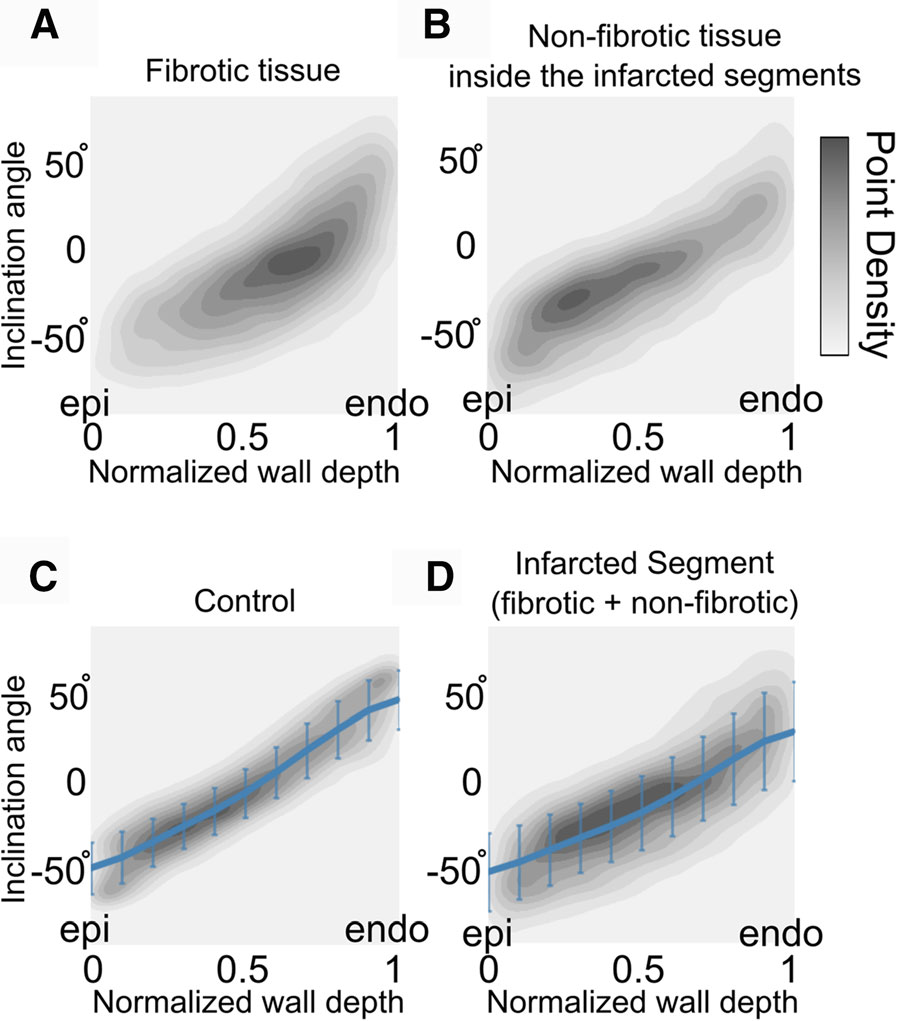
Average inclination angle profiles as a function of normalized wall depth. In each panel, voxel data corresponding to different tissue types are aggregated (**A**-**D**), with gray-maps representing data point density (*linear colorbar*). In **C** and **D**, averages of inclination angles at different wall depths are represented by blue lines (*error bars denote standard deviation*)

When the inclination angle of all voxels, fibrotic (n ~ 6.5 × 10^5^), non-fibrotic inside the infarcted segments (n ~ 1.4 × 10^6^), and control (n ~ 8.6 × 10^5^) were plotted together as a function of *normalized* wall depth, an interesting trend emerged: the fibrotic voxels tended to cluster within the endocardial half of the wall (Fig. 7A) and non-fibrotic voxels inside the infarcted segments clustered within the epicardial and midwall portion of the wall (Fig. 7B). Importantly, the average transmural epi-to-endo profile of inclination angle within the infarcted segments (Fig. 7D) was very similar to that of the control segments (Fig. 7C), indicating preservation of the underlying orientation within the infarcted segments. Further, the profile in the infarcted segments demonstrated a higher variance around the average at each point across the wall, and had a slightly lower inclination angle at the endocardium in comparison to the corresponding plot from control segments (Fig. 7C, D).

### Diffusion eigenvector orientation in the infarcted human heart

**Fig. 8:**
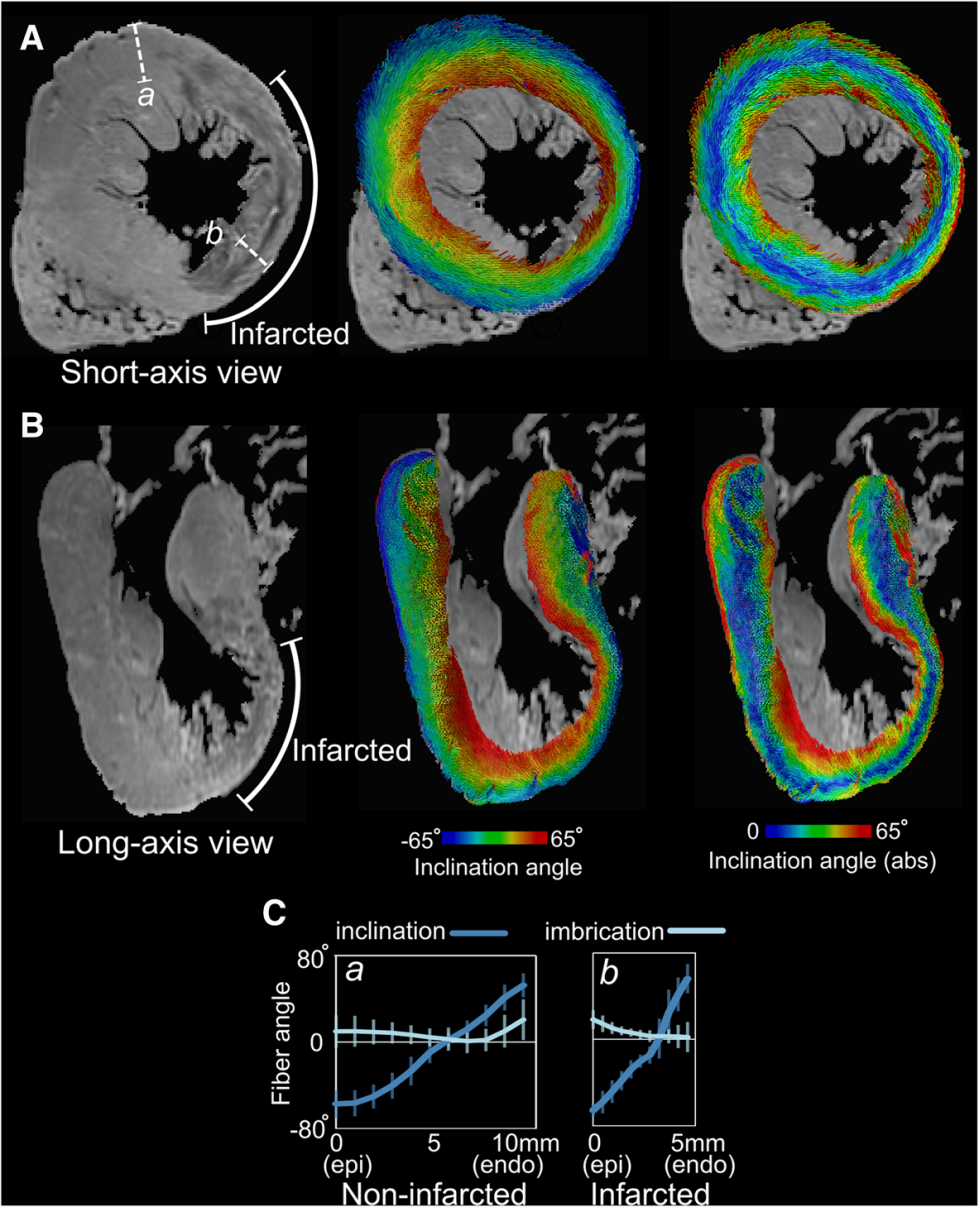
Fiber visualization in the human left ventricle (*LV*) with myocardial infarction. **A** short-axis view and **B** long axis view of the non-diffusion-weighted image (*left*), and eigenvector visualizations color-coded with inclination angle (*middle*) and absolute value of inclination angle (*right*). The infarcted wall is identifiable by the wall thinning at the LV anterioapical region as observed in the non-diffusion-weighted images. **C** Transmural angle profiles measured in two segments from non-infarcted and infarcted regions (*a* and *b* in panel **A** *left*). The local wall thickness in these two segments is ~10 mm and ~5 mm, respectively

Figure 8 presents a short-axis (A) and a long-axis (B) view of the non-diffusion-weighted image (left) as well as the primary eigenvector visualization (middle and right) in this heart. The infarcted area is identifiable by the significant wall thinning at the anteroapical LV region (left panels). As seen in the middle and right panels, the diffusion eigenvectors inside the thinned wall of the infarct demonstrate preservation of the original epi-to-endo transmural fiber angle pattern, but with a higher gradient. These observations are further shown in the angle profiles calculated from two segments, one inside and another outside the infarct (Fig. 8C). The finding of preservation of the primary eigenvector orientation in the infarcted wall in this human heart is consistent with that from the infarcted porcine hearts.

## Discussion

In this work, we studied chronic MI microstructural remodeling in eight porcine hearts and in a human heart non-destructively at submillimeter resolution. We examined the hearts’ fibrous structure using a customized DTI sequence on a clinical scanner (to accommodate the large specimen) that allowed acquisition of data over long scanning times with high image stability [21]. This, in addition to the high SNR and low artifact characteristics of the 3D spin echo sequence used here, resulted in high image quality and high spatial resolution. By combining this technique with high-resolution LGE imaging, we were able to provide reconstructions of both fiber architecture and scar distribution in infarcted hearts with an unprecedented level of detail. The submillimeter voxel size of the data (voxel volume: 0.432 mm^3^) resulted in an average of 8 voxels across the infarcted wall, allowing for the characterization of the structural remodeling in the zone of infarct, a task that has previously been particularly challenging for DTI due to significant infarct wall thinning and limited image resolution [14]. Importantly, it enabled us to systematically quantify the transmural pattern of diffusion eigenvector orientation in the porcine infarcts and to study the association between the level of structural remodeling and the extent of the infarct. Finally, we applied this technique to a human heart specimen to assess the remodeling at the thinned infarcted wall in the human heart.

The measurement of diffusion scalars in infarcted porcine hearts demonstrated an average of 43% increase in MD and a 35% decrease in FA inside the scar. Similar changes in MD and FA have been reported in DTI studies of myocardial infarcts in various species [11, 12, 14, 25, 26]. The increase in MD is indicative of a less restricted diffusivity and hence, of a larger diffusion volume for water molecules inside the scar. Myocyte death and subsequent collagen deposition following MI could explain this increase in diffusion volume. The same changes lead to alterations in the relative degree of diffusion anisotropy in the tissue, and therefore could be a factor in the reduction of FA. In addition, the dispersion of fiber angles (whether referring to the myofibers or collagen fibers in the infarct) within a voxel, either coherent or incoherent, could alone lead to a decrease in the measured FA due to an averaging effect. While coherent fiber dispersion exists in a normal LV due to the epi-to-endo change in inclination angle, an increase in this dispersion due to an increase in the transmural gradient of fiber angles (such as that shown in Fig. 5A) could, in principle, reduce FA. This would be particularly true for lower image resolution estimates of FA. In addition to this, incoherent dispersion of fibers at the infarct (fiber disarray) has been shown to play significant role in the reduction of FA. Using histological characterization of rat infarcts, Chen et al. [25] found a good correlation between the amount of fiber disarray and the decrease in FA value. The extent to which factors like fiber dispersion and changes in tissue composition contribute to the measured FA value is unknown and requires further investigation. In our study, despite the lower anisotropy inside the infarct, the measured FA in the majority of infarct regions was greater than that of the isotropic water, which revealed that the chronic scar was mostly comprised of anisotropic structures. This is in agreement with previous histological studies demonstrating that scarred tissue in the porcine heart has a high content and high alignment of collagen [27]. We also found highly aligned collagen bundles from histological imaging of the infarcted region (Additional file 1: Figure S5), likely constituting the main source of anisotropy in the infarct.

The presence of diffusion anisotropy allowed us to employ the diffusion eigenvectors to investigate the anisotropic organization of the collagenous scar. We found that, on average, the orientation of the eigenvectors at the infarcted segments followed the pattern of the original fiber orientation, i.e. left-handed fibers at the epicardium to right-handed fibers at the endocardium. This result is consistent with previous findings in rats [25] and sheep [16] but is contrary to findings in another study in rats, which reported severe perturbation of eigenvector orientation in the infarct [28]. In the current study, we provided further evidence for the preservation of primary eigenvector angles and the increase in the slope of transmural angle profile in chronic porcine infarcts and in a human infarct. Despite the preservation of average fiber angles at the infarcted segments and the local collagen fiber alignment in the histology images, we also found that the average intervoxel incoherency of the inclination and imbrication angles in the infarct is higher than the control segments. Monte-Carlo analysis (see Additional file 1: Figure S1B) showed that this increase in the incoherency could not completely stem from the increase in the uncertainty in the estimation of primary eigenvector resulting from reduction in FA in the infarcted region, suggesting the presence of a microstructural basis. Finally, the investigation of structural remodeling at different levels of wall thickness and of the degree of scar transmurality in our study indicates that the inclination angle range is primarily preserved in the infarcted regions and that the increase in the slope is mainly due to wall thinning.

In this study, we found an increase in the proportion of left-handed primary eigenvectors inside the infarcted segments of porcine hearts, which had also been observed in two previous studies of human hearts [13, 18]. We can speculate that this could be due to non-uniform wall thinning across the wall; the sub-endocardial wall and midwall are more likely to be affected by ischemia and undergo more thinning. In support of this, we indeed observed non-uniform distribution of scar across the wall, with fibrosis more concentrated at the sub-endocardial and midwall sub-layers of the infarcted wall (Fig. 7A). The distribution of primary eigenvector angle at the infarcted segments could have also been affected by the patterns of mechanical stress and strain during the process of remodeling. In general, despite slight differences in the shapes of the inclination angle profiles, there was a remarkable similarity between the angle profile trends in control and in infarcted segments. As a proof of concept, preservation of the trend of inclination angle profile across the region of infarct was also observed in the thinned wall of the single human heart in the study.

After MI, the organization of collagen fibers in the scar is influenced by structural and mechanical factors [29]. The original extracellular matrix (ECM) acts as scaffold for the deposition of the new collagen [29]. In addition, the passive stretch of the infarcted tissue by the surrounding myocardium could influence the collagen alignment and hence the anisotropic structure of the scar [30]. The extent to which these factors contribute to scar structure is not known. Our observations in porcine and human ventricles suggest that the existing ECM might play a significant role in the alignment of collagen fibers, as the general transmural patterns of primary diffusion eigenvector angles are preserved inside the scar. This information could be important to therapeutic approaches in the treatment of the infarct, such as tissue engineering and regenerative medicine, as ECM orientation is likely to determine the orientation of regenerated myocytes [31].

Accurate structural data is essential for the construction and validation of whole-heart computational models [32–35]. These models have enabled advancements in the understanding and treatment of cardiac dysfunction. DTMRI data derived from normal hearts have been utilized by many groups to accurately represent the fiber orientation in cardiac models [36]. However the paucity of data from infarcted hearts has prevented modeling research from fully evaluating the effect of structural remodeling on electrical and mechanical dysfunction in whole-heart cardiac models, particularly in large animal and human hearts [36]. The data presented here provide unprecedented detail about myofiber orientation and scar geometry in infarcted hearts. These data could be employed to construct high-resolution image-based models to investigate the mechanistic links between infarct-related arrhythmias and structural remodeling, including fibrosis distribution and fiber arrangement [37]. In addition, the 3D information regarding the collagen fiber orientation in the intact scar as provided here could improve the accuracy of the modeling approaches aimed at understanding the mechanical role of the passive scar on post-MI ventricular function [38–40]. This could ultimately lead to optimal design of therapies aimed at modifying the electromechanical properties of the infarct [31, 41, 42]. Furthermore, patient-specific models are being constructed from clinical images with clinical applications such as patient arrhythmia risk stratification and optimal treatment planning for rhythm disorders [43, 44]. A complete picture of the high-resolution structural detail in the infarct will assist in making the appropriate assumptions when creating patient-specific models from lower resolution clinical images [33]. The integration of realistic fiber orientation in infarcted hearts, as obtained in this study, will enhance the accuracy of these clinical modeling efforts.

### Limitations

This study has several limitations. First, the LAD reperfusion infarct induction protocol in porcine hearts may render our findings not entirely applicable to all chronic infarctions in the human. Second, although the main findings from the analysis of the porcine hearts regarding the preservation of eigenvector orientation and the increase in the transmural slope were in agreement with the those of the human heart in this study, the single human specimen was not sufficient for a full characterization of infarct remodeling in human hearts and was only a simple existence demonstration. Future studies are needed to investigate this in a larger number of samples. Also since this specimen did not undergo LGE imaging, we were not able to accurately delineate the infarcted area and provide systematic analysis of the remodeling in this heart. Third, despite submillimeter resolution of the imaging with average of ~8 voxels across the wall in the infarct, we excluded the regions with wall thickness less than 2 mm to ensure reliable measurement of transmural angle profile with at least ~4 voxels across the wall; this excluded around 10% of the infarcted wall. Finally, while the fixation process could have affected the baseline values of diffusivity such as FA and MD in our study [45], it has been shown that it does not change the eigenvector orientation significantly after infarction [45]. So we do not believe it could have affected the main findings of the study.

## Conclusions

The application of 3D DTI and LGE-CMR revealed the fiber orientation and scar geometry in infarcted porcine hearts at an unparalleled resolution and SNR. The results demonstrated preservation of eigenvector orientation, with a higher transmural gradient of inclination angle at the thinned wall of infarct. Detailed information of post-infarction remodeling obtained in this study could pave the way for generation of accurate whole-heart models of infarcted hearts to investigate the mechanistic links between the structure and electromechanical function, and thus may lead to improvements in therapies after myocardial infarction.

## Additional file

Additional file 1: Supplemental Methods and Results. **Supplemental Methods:** SNR Measurement, Effect of SNR on the estimation of FA and principal eigenvector (**Figure S1**), Calculation of FA in isotropic liquid (**Figure S2**), Quantification of local angle incoherency, **Supplemental Results:** Pair-wise distributions of primary and secondary eigenvalues (**Figure S3**), Preservation of papillary muscles at the infarcted area (**Figure S4**), Histology in a section of infarcted wall using Trichrome staining (**Figure S5**), Structural metrics in infarcted and control segements at different levels of scar transmurality (**Table S1**). (PDF 5478 kb)

## Abbreviations

3D: Three-dimensional
DTI: Diffusion tensor imaging
DTMRI: Diffusion tensor magnetic resonance imaging
ECM: Extracellular matrix
FA: Fractional anisotropy
LAD: Left anterior descending
LGE: Late gadolinium enhancement
LH: Left-handed
LV: Left ventricle
MD: Mean diffusivity
MI: Myocardial infarction
MRI: Magnetic resonance imaging
RH: Right-handed
SNR: Signal-to-noise ratio
TE: Echo time
TR: Repetition time
