# Supplementary material for: Submillimeter diffusion tensor imaging and late gadolinium enhancement cardiovascular magnetic resonance of chronic myocardial infarction
Source: J Cardiovasc Magn Reson. 2017 Jan 11;19:9. doi: 10.1186/s12968-016-0317-3 (PMC5264305; doi:10.1186/s12968-016-0317-3)
Supplement: Additional file 1: — Supplemental Methods and Results. Supplemental Methods: SNR Measurement, Effect of SNR on the estimation of FA and principal eigenvector (Figure S1), Calculation of FA in isotropic liquid (Figure S2), Quantification of local angle incoherency, Supplemental Results: Pair-wise distributions of primary and secondary eigenvalues (Figure S3), Preservation of papillary muscles at the infarcted area (Figure S4), Histology in a section of infarcted wall using Trichrome staining (Figure S5), Structural metrics in infarcted and control segements at different levels of scar transmurality (Table S1). (PDF 5478 kb) [file 12968_2016_317_MOESM1_ESM.pdf]

Supplementary Methods

SNR Measurement

To measure the image SNR in the infarcted and control hearts, one representative control and one infarcted heart were chosen and SNR values were measured in the non-DW images, as follows: in 5 slices selected uniformly from apex to base, regions of interests (ROI) were drawn with equal areas both inside the myocardium and in the background with close to zero intensity (for the infarcted heart, ROIs were drawn both in the remote non-infarcted as well as in the infarcted region). At each slice, the SNRs were estimated by calculating the ratio of the mean foreground ROI intensity (control, non-infarcted or infarcted) to the standard deviation of the intensity of the corresponding background ROI, and multiplying the ratio by 0.655, to take into account the Rayleigh distribution of the background noise:

$$SNR = 0.655 \frac{mean(I_{foreground})}{std(I_{background})} \quad (S1)$$

The mean SNR was 122 ± 11 for control, 184 ± 13 for infarcted, and 113 ± 15 for non-infarcted remote regions.

Effect of SNR on the estimation of FA and principal eigenvector

The dependency of the uncertainty of FA and eigenvector estimations on SNR have been shown previously in the brain studies [1, 2]. Here we performed a similar Monte Carlo simulation [1] to investigate the effect of SNR on the estimation of FA and principal

eigenvector in control and infarcted regions of the heart in our study. First, eigenvalues (e1,e2,e3) of representative voxels from each of the control and infarcted regions were calculated. Then for a given set of eigenvalues, 15 synthetic diffusion-weighted signals were generated with the b-value and gradient table the same as the diffusion acquisition in our study, assuming the eigenvector is oriented along the x-axis. To model the effect of thermal MR noise on the data, the original synthetic DW and b0 data were considered as real-values signals and were added by a complex-valued noise signal with the real and imaginary components having a Gaussian distribution of zero mean and standard deviation σ. The magnitudes of the final complex noisy signals were computed (total of 15 DW + 1 b0 noisy signals), and the tensor reconstruction was performed on the set of noisy images to obtain the principal eigenvector and FA. This process was repeated N = 10000 times for a given noise-level (σ). The σ was varied to generate different values of SNR (ranging from 5 to 200). SNR was calculated according to Equation S1 by calculating the corrected ratio of the mean intensity of the generated b0 images to the standard deviation of the magnitude of the noise signal. For a given value of SNR, the mean and standard deviation of FA was calculated and plotted as a function of SNR (Figure S1). In addition, the uncertainty of the principal eigenvector was measured by calculating the angle dispersion (standard deviation) of the primary eigenvectors around the average (calculated using dyadic tensor approach [3] to handle the fact that the eigenvectors are defined along their axis). This value has been plotted as a function of SNR in Figure S1. The results demonstrate that at the operating SNRs in the normal and infarcted tissues (gray boxes on the plots), there is virtually no effect of SNR bias on the FA value. In addition, the difference between the uncertainty of the primary eigenvector

estimation (as quantified by angle STD -- panel B), is around 1-2 degrees between the infarcted and control regions at the operating SNRs of our imaging.

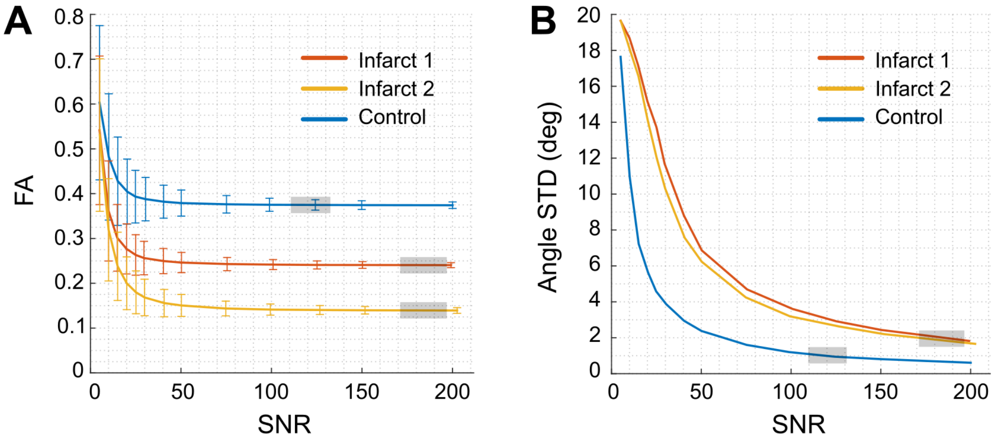

Supplementary Figure S1. Effect of SNR on the estimation of FA and principal eigenvector using Monte Carlo simulation. **(A)** FA vs. SNR, and **(B)** Uncertainty of angle estimation vs. SNR. The calculation is performed in two representative voxels from infarct and one from control regions. The gray boxes delineate the operating SNR in the control and infarcted tissues.

Calculation of FA in isotropic liquid

To calculate FA in an isotropic medium, a region of leftover blood/formaldeyde inside the LV chamber was manually selected comprising of ~1200 voxels. This region was selected from the same infarcted heart (and in the same scan) that was used to calculate SNR in the previous section. However, the isotropic region demonstrated a higher intensity than infarcted or non-infarcted regions (due to a high T2 value). The estimated SNR in this region was ~ 380. Figure S2,A demonstrates the distribution of FA in this isotropic medium ($FA_{iso} = 0.027 \pm 0.007$). Figure S2, B shows a similar simulation

results as the previous section, suggesting a negligible effect of SNR on the measured FA at the operating SNR in the isotropic liquid.

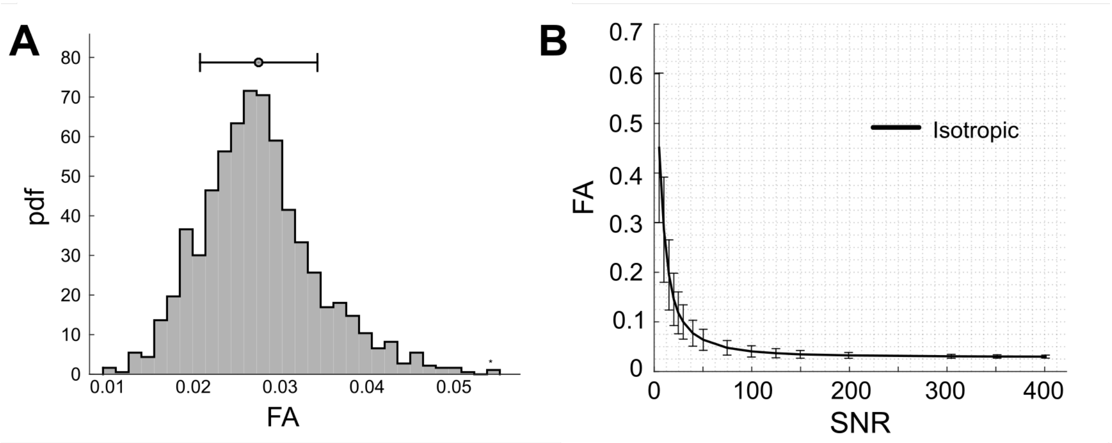

Supplementary Figure S2. Estimation of FA in isotropic region. **(A)** Distribution of measured FA in an region of interest in an isotropic liquid, and **(B)** Result of Monte Carlo simulations demonstrating the effect of SNR on the estimated FA in a voxel from the isotropic liquid.

Quantification of local angle incoherency

To quantify the local incoherency in the primary eigenvector arrangement, individual normal and infarcted LV segments were divided into 10 sub-segments equally spaced through the depth of the wall. The standard deviations of the inclination and imbrication angles in each sub-segment were calculated and the results were averaged over the 10 sub-segments, yielding two measures of fiber incoherency for each segment that together quantified the extent of local incoherency of the diffusion vector field:

$$incoherency_{\theta} = \frac{1}{L}\sum_{i=1}^{L} STD_i(\theta) \qquad (S2)$$

In which θ is either the inclination or the imbrication angle, *L=10*, and $STD_i(\theta)$ is the unbiased standard deviation of the angles within the *i*th sub-segment.

In comparison to direct calculation of local fiber angle gradient with a fixed kernel [4], the calculation of incoherency metrics, as presented here with the radial dimensions of the sub-segments normalized to wall thickness, is less sensitive to uniform changes in the transmural gradient of fiber angles arising purely from changes in local wall thickness.

Supplementary Results

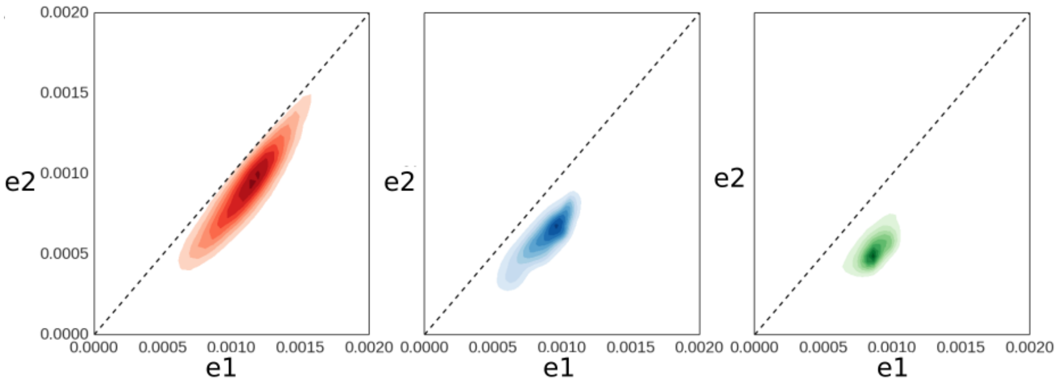

Supplementary Figure S3. Pair-wise kernel density estimation (KDE) for secondary (e2) vs. primary eigenvalues (e1) in fibrotic (left panel) and non-fibrotic (middle panel) tissues from infarcted hearts, and the normal tissue (right panel). Units are mm^2/s.

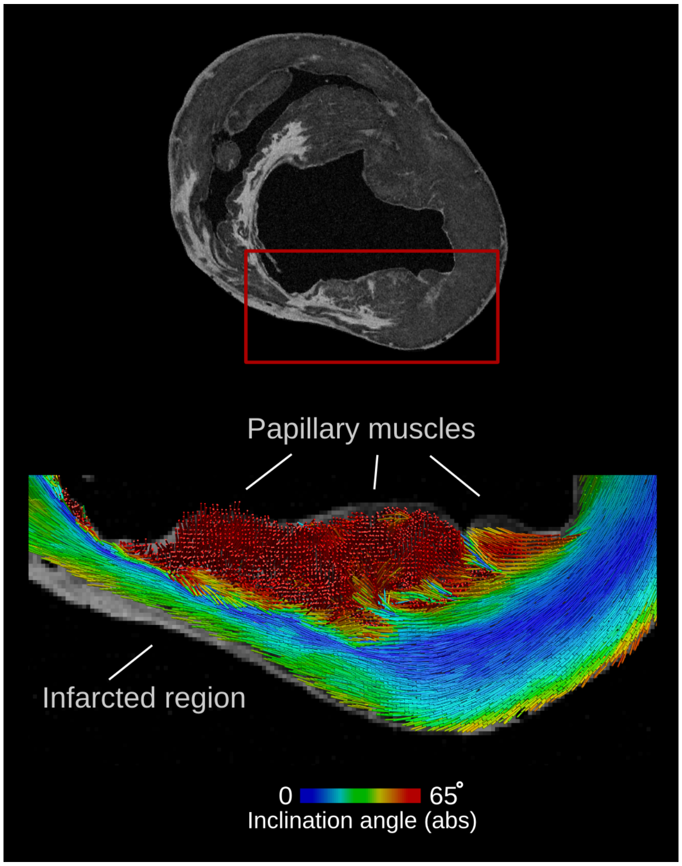

Supplementary Figure S4. Preservation of papillary muscles at the infarcted area. The view is similar to Figure 1F,G. Top: A short-axis slice of LGE, Bottom: primary eigenvector visualization, color-coded by inclination angle.

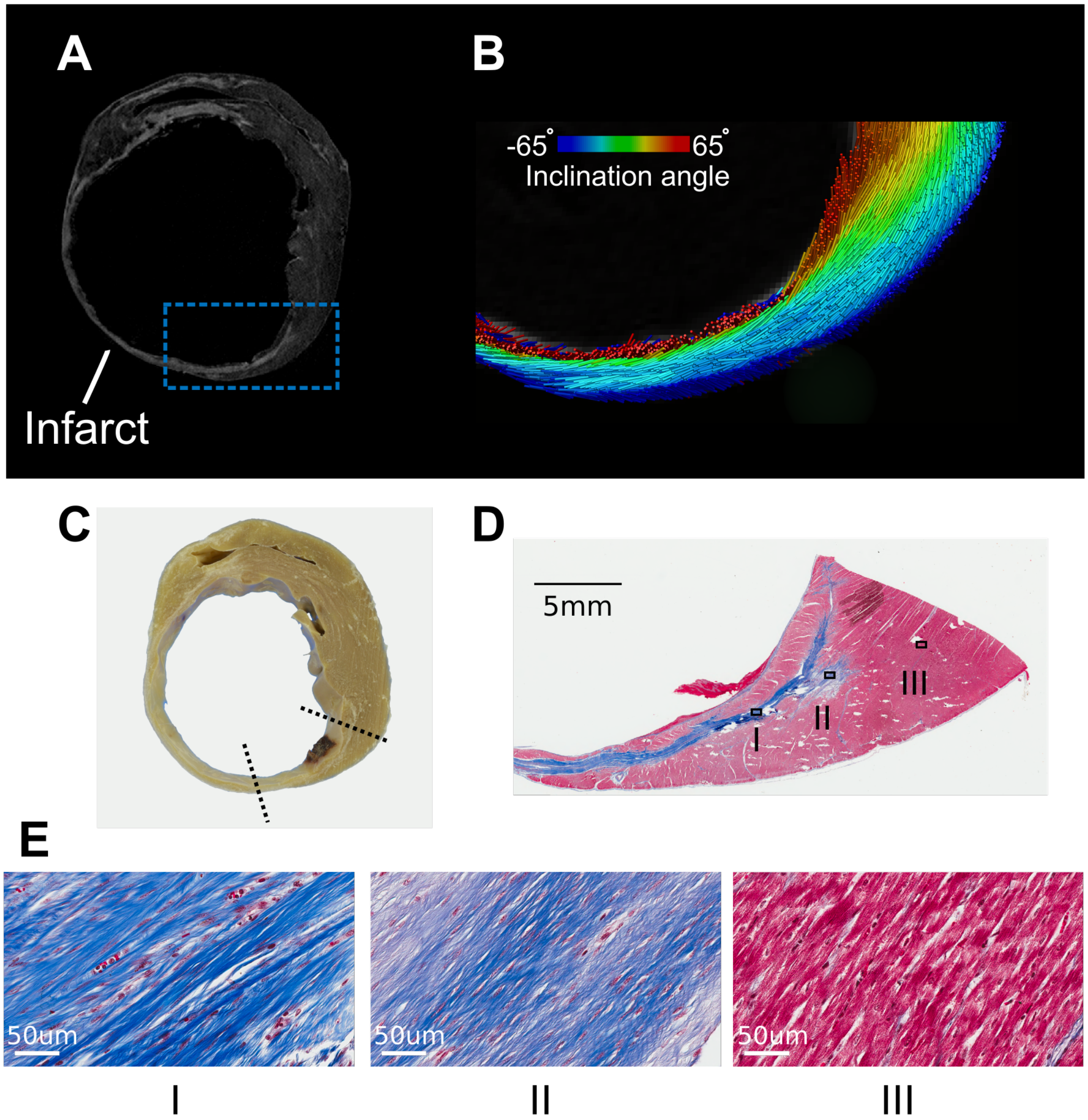

Supplementary Figure S5. Histology in a section of infarcted wall using Trichrome staining. The infarct is 4 months old. **(A)** A slice of LGE image delineating the hyper-enhanced infarct area. **(B)** Visualization of primary eigenvector in a region of the wall (blue box in panel A) color-coded by inclination angle. **(C)** A sliced section of the heart with a similar view as panel B. **(D)** Trichrome staining histology of the region highlighted in panel C. The blue demonstrates the collagenous scar. Three regions have been selected in infarcted and control area **(E)** 40X magnification of the regions selected in (D). I and II demonstrate the alignment of collagen bundles in the fibrotic area. III represents the myocardial tissue in a non-infarcted region.

Supplementary Table S1. Structural metrics in infarcted and control segements at different levels of scar transmurality.

Quantity	Infarcted Segments						Control Segments
	Scar transmurality: [0-0.33]		Scar transmurality: [0.34-0.66]		Scar transmurality: [0.67-1]		
	Mean ± STD	P-value	Mean ± STD	P-value	Mean ± STD	P-value	Mean ± STD
Wall thickness (mm)	7.3 ± 1.4	0.610	4.7 ± 1.0	0.017	3.9 ± 0.8	0.007	7.0 ± 0.9
Scar transmurality	0.13 ± 0.03	NA	0.51 ± 0.02	NA	0.79 ± 0.05	NA	NA
Inclination angle range (°)	104.7 ± 11.3	0.126	112.1 ± 13.5	0.734	116.8 ± 11.9	0.734	112.5 ± 6.8
Slope (°/mm)	14.0 ± 4.9	0.308	24.1 ± 9.3	0.126	29.7 ± 9.8	0.042	15.7 ± 1.1
Intercept (°)	-55.2 ± 7.9	0.734	-62.6 ± 13.2	0.234	-65.9 ± 10.4	0.042	-54.0 ± 5.3
r^2	0.74 ± 0.06	0.006	0.66 ± 0.08	0.007	0.68 ± 0.04	0.007	0.93 ± 0.03
Imbrication angle mean (°)	1.11 ± 10.42	0.610	3.50 ± 11.84	0.865	0.18 ± 10.31	0.308	0.84 ± 1.47
Inclination incoherency (°)	8.81 ± 1.46	0.007	10.67 ± 1.56	0.007	11.15 ± 1.21	0.007	5.63 ± 0.56
Imbrication incoherency (°)	7.90 ± 1.66	0.017	9.37 ± 1.97	0.007	8.82 ± 1.46	0.007	4.83 ± 0.58
LH-ratio	0.48 ± 0.09	0.089	0.52 ± 0.10	0.027	0.53 ± 0.05	0.007	0.39 ± 0.05
Circumferential-ratio	0.32 ± 0.07	0.865	0.25 ± 0.09	0.234	0.22 ± 0.06	0.042	0.30 ± 0.04
RH-ratio	0.20 ± 0.05	0.017	0.22 ± 0.08	0.126	0.25 ± 0.08	0.308	0.31 ± 0.03

Wilcoxon rank-sum test was performed between the infarcted (n=8) and control (m=4) hearts.

References

[1] C. Pierpaoli and P. J. Basser, "Toward a quantitative assessment of diffusion anisotropy.," *Magn. Reson. Med.*, vol. 36, no. 6, pp. 893–906, Dec. 1996.

[2] J. A. D. Farrell, B. A. Landman, C. K. Jones, S. A. Smith, J. L. Prince, P. C. M. Van Zijl, and S. Mori, "Effects of Signal-to-Noise Ratio on the Accuracy and Reproducibility of Diffusion Tensor Imaging – Derived Fractional Anisotropy , Mean Diffusivity , and Principal Eigenvector Measurements at 1 . 5T," vol. 767, pp. 756–767, 2007.

[3] D. K. Jones, “Determining and visualizing uncertainty in estimates of fiber orientation from diffusion tensor MRI.,” *Magn. Reson. Med.*, vol. 49, no. 1, pp. 7–12, Jan. 2003.

[4] G. J. Strijkers, A. Bouts, W. M. Blankesteijn, T. H. J. M. Peeters, A. Vilanova, M. C. van Prooijen, H. M. H. F. Sanders, E. Heijman, and K. Nicolay, “Diffusion tensor imaging of left ventricular remodeling in response to myocardial infarction in the mouse.,” *NMR Biomed.*, vol. 22, no. 2, pp. 182–90, Feb. 2009.
